# The hemostatic molecular mechanism of Sanguisorbae Radix's pharmacological active components based on HSA: Spectroscopic investigations, molecular docking and dynamics simulation

**DOI:** 10.1016/j.heliyon.2024.e37020

**Published:** 2024-08-28

**Authors:** Fei Xu, YuQing Shen, ZhiQiang Pan, Xuan Zhou, Wei Gu, Jie Dong, Shaoping Yin, ShengJin Liu, Ming Xu, Baoduan Chen

**Affiliations:** a National Key Laboratory on Technologies for Chinese Medicine Pharmaceutical Process Control and Intelligent Manufacture, Nanjing University of Chinese Medicine/Jangsu Kanion Pharmaceutical Co., Ltd., Nanjing, 210023, China; b The Second People's Hospital of Taizhou City, Taizhou, 225500, China; c Jiangsu Provincial Engineering Research Center of TCM External Medication Development and Application, Nanjing University of Chinese Medicine, Nanjing, 210023, China; d School of Pharmacy, Nanjing University of Chinese Medicine, Nanjing, 210023, China; e Jiangsu Key Laboratory of Chinese Medicine Processing, Nanjing, 210023, China; f Jiangsu Sunan Pharmaceutical Group Co., Ltd., Zhenjiang, 212400, China; g School of Elderly Care Services and Management, Nanjing University of Chinese Medicine, 210023, China

**Keywords:** Sanguisorbae Radix phenolic acid, Haemostasis, HSA, Multi-spectroscopy, Molecular simulation

## Abstract

The interactions between human serum albumin (HSA) and the hemostatic components of the Chinese medicine Sanguisorbae Radix (SR), specifically phenolic acid compounds such as caffeic acid (CA), ferulic acid (FA) and their 1:1 mixture (1:1) were studied to investigate the molecular mechanism underlying the hemostatic effect of SR. Network pharmacology combined with the experimental and computational data revealed that HSA is one of the hemostatic targets to SR phenolic acids. SDS-PAGE and multi-spectroscopy demonstrated that the phenolic acids bind to the Sudlow site I on HSA, altering its structure and influencing its migration velocity. There is an observed synergistic effect upon the mixture of CA and FA. Quantum chemistry, molecular docking, and molecular dynamics simulations indicate that the binding of phenolic acids to HSA is stable, and variations in binding efficiency are associated with the hydrophobicity of the substituent at the C3 position of the side chain, and also, the key amino acids and functional groups for hemostasis of SR were identified, along with the active sites that contribute to the synergistic enhancement by phenolic acids.

## 1 Introduction

Hemorrhagic conditions are among the most common and variable clinical symptoms, manifesting in various forms such as hematemesis, epistaxis, and hematochezia [1]. Currently, the prevalent hemostatic pharmaceuticals include agents like pituitary posterior lobe extracts, adrenochrome monosemicarbazone, and tranexamic acid, which often induce adverse reactions such as nausea, vomiting, dizziness, and tinnitus [2]. In recent years, traditional Chinese medicine has garnered widespread attention due to its low toxicity, effective treatment outcomes, and lack of drug resistance [3]. Research into Chinese medicines with hemostatic properties and their active components is not only beneficial for elucidating the mechanisms by which these medicines achieve hemostasis but also advantageous for the development and utilization of traditional Chinese medicine.

Sanguisorbae Radix (SR) is the dried root of the Rosaceae family plant *Sanguisorba officinalis* L, and is commonly employed as a traditional Chinese medicine hemostatic agent in clinical practice [4]. Modern research has shown that SR increases the content of erythrocytes in the blood, leading to the phenomenon of axial accumulation and a consequent reduction in the thickness of the peripheral plasma layer. This results in an elevated whole blood concentration and a deceleration of blood flow velocity, which is conducive to the anticoagulant function of platelets, thereby exerting hemostatic medicinal effects. However, the molecular mechanism of this hemostasis has not yet been reported [[5], [6], [7]].

The hemostatic active components in SR are phenolic acid compounds, which mainly include caffeic acid (CA), ferulic acid (FA), ellagic acid (EA), gallic acid (GA), and catechin (C) [8,9]. In this study, network pharmacology combined with literature analysis was utilized to identify human serum albumin (HSA) as one of the functional proteins involved in the hemostatic activity of phenolic acids in SR. HSA has a regulatory effect on blood agglutination and participates in the process of coagulation and haemostasis. It can change the way of platelet aggregation by combining with nitric oxide (NO) and affect the function of platelets [[10], [11], [12]]. At the same time, HSA is the most abundant carrier protein in plasma, with stability and transport properties. It can bind with active ingredients and transport to the target organ to exert the drug effect. The binding of drug molecules to HSA has a great influence on their metabolism and distribution in the body [[13], [14], [15]]. HSA has higher solubility, negative charge, and better stability than other proteins, which makes it an ideal model protein for the study of the interactions between biomolecules and small drug molecules and in vivo metabolism [16].

Therefore, in this study, we selected CA and FA, the representative components with structural regularity in SR phenolic acid and designed a mixture of CA:FA at 1:1 ratio (1:1) based on the content of both in SR herbs, which are the main research objects [17]. Sodium dodecyl sulfate-polyacrylamide gel electrophoresis (SDS-PAGE), multiple spectroscopy methods (ultraviolet and visible spectroscopy, fluorescence quenching, simultaneous fluorescence, and circular dichroism), quantum chemistry, molecular docking, and visualized molecular dynamics were combined to characterize the interaction between SR phenolic acid and HSA at the atomic level. The binding strength and binding sites of FA, CA, and their 1:1 ratio with HSA were assessed, characterizing the alterations in the secondary structure of HSA. Molecular docking, MD and quantum chemistry were used to further quantify the interactions and visualized the expression of non-covalent interactions, among others. This study explored the molecular mechanism of SR hemostasis by describing the interaction between the hemostatic active ingredients of SR and HSA, which has not yet been reported in the literature. The results of this study provide a theoretical basis and clinical guidance for the development and application of hemostatic traditional Chinese medicines, as well as references for the binding and transport modes of drugs and carrier proteins.

## 2 Materials and methods

### 2.1 Materials

**Fig. 1:**
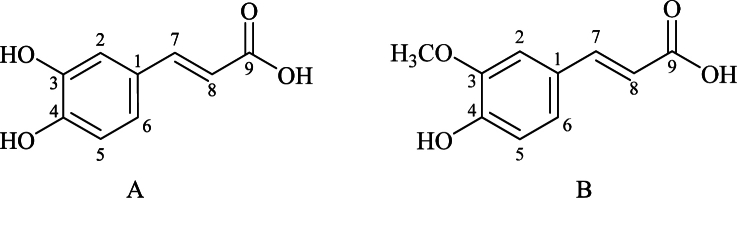
Molecular structures of SR phenolic acid. A. CA, B. FA.

The standard substances of CA and FA were obtained from Chengdu Must Bio-Technology Co., Ltd., with a purity of 98 %, and their structures are illustrated in Fig. 1. HSA was sourced from Sigma, USA. Anhydrous ethanol was provided by Nanjing Chemical Reagent Co., Ltd. The SDS-PAGE protein loading buffer (5 × loading buffer) was procured from Shanghai Beyotime Biotechnology Co., Ltd. Glycine (Gly) was from Beijing Solarbio Science & Technology Co., Ltd. Acrylamide and N,N′-methylenebisacrylamide were supplied by Shanghai Macklin Biochemical Co., Ltd. The 4 × SDS-PAGE stacking gel buffer was from Beijing Solarbio Science & Technology Co., Ltd. Ammonium persulfate was from Aladdin Bio-Chem Technology Co., Ltd. (Aladdin). N,N,N′,N′-Tetramethylethylenediamine (TEMED) was from Sigma, USA. The pre-stained protein marker was from Nanjing Jiancheng Bioengineering Institute. Coomassie Brilliant Blue R250, the Coomassie Blue staining and destaining solution (conventional method) and the red blood cell lysis buffer were from Shanghai Beyotime Biotechnology Co., Ltd. Tris-(hydroxymethyl)-aminomethane (Tris) was from Beijing Solarbio Science & Technology Co., Ltd., and it was mixed with hydrochloric acid (HCl) to prepare a pH 7.4 Tris-HCl buffer solution (0.50 mol L^−1^).

Ibuprofen, with a purity of 98 %, was from Shanghai Macklin Biochemical Co., Ltd. Warfarin, also at a purity of 98 %, was from Shanghai Aladdin Bio-Chem Technology Co., Ltd. The methanol used was of chromatographic grade, sourced from Fisher Scientific. Sodium chloride was from Sinopharm Chemical Reagent Co., Ltd., and the water used in experiments was ultrapure water.

### 2.2 Instruments

The Cary-5000 UV–Vis spectrophotometer from Varian, USA; the LS55 fluorescence spectrometer from PerkinElmer, USA; the analytical balance from Shanghai Shangping Instruments Co., Ltd.; the MX-S Vortex Mixer from SCILOGEX, USA; the PHS-3C pH meter from Shanghai Yidian Scientific Instrument Co., Ltd.; the Jasco-810 spectrophotometer from Photophysics, UK (Easton, MD, USA); the electrophoresis system from Bio-Rad, USA; the ambient centrifuge from Thermo Fisher Scientific, USA; the Dry-Bath and the shaker from SCILOGEX, USA; the gel imaging and analysis system from Shanghai TANON Science&Technology Co., Ltd..

### 2.3 Animals

ICR male mice, weighing approximately 20 g, a cohort of five, were procured from Jiangsu Huachuang Xinuo Pharmaceutical Technology Co., Ltd., with the license number SYXK (Su) 2023-0077. All experimental procedures were approved by the Animal Ethics Committee of Nanjing University of Chinese Medicine.

### 2.4 Methods

#### 2.4.1 Solution preparation

Sample solution preparation: Accurately weigh the required amounts of CA and FA standards and dissolve them in methanol to prepare monomeric solutions (5.12 × 10^−4^ mol L^−1^). Dilute with Tris-HCl buffer solution (0.50 mol L^−1^, pH = 7.4) to obtain CA and FA solutions with a concentration of 1.57 × 10^−4^ mol L^−1^, and store at 4 °C.

HSA solution preparation: Measure an appropriate quantity of HSA and dissolve it in Tris-HCl buffer solution. Mix thoroughly to prepare a solution of HSA at a concentration of 10.8 μmol L^−1^, and store in a refrigerator at 4 °C.

#### 2.4.2 Network pharmacology

(1) Collection and screening of SR targets [[18], [19], [20]].

The 3D structure files in SDF format of the hemostatic active phenolic acid compounds of SR (CA, FA, EA, GA, C) were obtained from the PubChem database (http://pubchem.ncbi.nlm.nih.gov). These files were then imported into the PharmMapper database (http://www.lilab-ecust.cn/pharmmapper) to identify potential targets for the phenolic acids in SR. The standard identifiers for the target proteins were retrieved from the Uniprot database (https://www.uniprot.org/), thereby establishing a common target database for the principal components of SR phenolic acids.

(2) Identification of hemostatic disease targets [21].

Using “Bleeding” as a keyword, a search was conducted on the GeneCards database (https://www.genecards.org/) to find target proteins related to hemorrhagic diseases. The intersection targets between SR phenolic acids and hemorrhagic diseases were extracted using the R programming language.

The phenolic acid targets of SR and hemostatic action were imported into Cytoscape 3.9.1 software for visualization. Each target protein and component were set as nodes, with edges between connected nodes, to construct a network of interaction targets. Within this visualized network, potential target proteins for the treatment of hemorrhagic diseases by SR were identified.

(3) Construction of the protein-protein interaction (PPI) network [22,23].

The potential hemostatic target proteins of SR phenolic acids were imported into the STRING database for retrieval, specifying the research species as “*Homo sapiens*.” PPI relationships with a confidence score greater than 0.5 were selected, nodes not connected within the network were not displayed, and other parameters were set to default. The resulting PPI network depicting protein interactions was then imported into Cytoscape 3.9.1 for topological attribute analysis.

#### 2.4.3 SDS-PAGE [[24], [25], [26], [27]]

(1) Erythrocyte lysis

Blood was drawn from the orbital plexus of ICR mice and collected in tubes containing the anticoagulant heparin. The samples were then centrifuged at 500 g for 5 min, the supernatant was discarded, and 6–10 vol of erythrocyte lysis buffer were added. The mixture was gently vortexed to ensure homogeneity and lysed for 2 min, followed by a further centrifugation for 5 min to discard the red supernatant. The pellet was resuspended in an appropriate volume of PBS, centrifuged for 3 min, and the supernatant was discarded. This washing step was repeated twice, with the volume of the washing solution being five times that of the cell pellet.

(2) Drug treatment and extraction of serum albumin

The prepared whole blood samples were randomly divided into four groups: blank plasma group, CA, FA, and a CA:FA = 1:1 combination group. After respective treatments, the samples were incubated in a 37 °C metal bath for 1 h. An appropriate amount of 5 × loading buffer was added, followed by heating in a 100 °C metal bath for 5 min. The samples were then aliquoted and stored at −80 °C for future use.

(3) Protein electrophoresis experiment

A 10 % sodium dodecyl sulfate-polyacrylamide gel was prepared. A volume of 30 μL of HSA solution, at a concentration of 0.033 μg/μL, was loaded onto the gel as the HSA blank group. The prepared protein samples from step (2) were loaded in the sequence of blank plasma group, CA, FA, and 1:1, with 30 μL per well. Pre-stained protein markers were added to the edge wells at 2 μL each. After loading the five groups of samples: HSA blank group, blank plasma group, CA, FA, and 1:1, an appropriate amount of 1 × running buffer was added to the electrophoresis tank. The conditions for the sample running through the stacking gel were 60 V for 30 min, and for the separating gel, 95 V for 60 min.

After electrophoresis, the gel was immersed in a sufficient volume of Coomassie Brilliant Blue staining solution, ensuring complete coverage of the gel. The gel was then gently agitated on a shaker at room temperature for 4 h before the staining solution was discarded. An adequate amount of destaining solution was added to fully cover the gel. The gel was agitated slowly on a shaker at room temperature for 4 h, with the destaining solution being changed three times during the process. After destaining was complete, the gel was preserved in water for imaging and photography.

#### 2.4.4 UV–Vis absorption spectroscopy [28,29]

2 mL of HSA solution was transferred into a 1 cm quartz cuvette, and five aliquots of 5 μL of CA solution, FA solution, and 25 μL of a 1:1 mixed solution were successively added. After each addition, the solution was thoroughly mixed to achieve phenolic acid to HSA molar ratios of 0, 0.74, 1.48, 2.22, 2.96, and 3.7, respectively. The solution was allowed to stand until the reaction is complete. The measurement conditions are as follows: scan the range from 200 to 300 nm with a scanning interval of 1.0 nm, using 0.50 mol L^−1^ Tris-HCl buffer solution (pH = 7.4) as the reference. The changes in the ultraviolet–visible absorption spectrum of the HSA solution were recorded.

#### 2.4.5 Fluorescence quenching method [30,31]

Sequentially introduced five batches of 5 μL CA solution, FA solution, and 25 μL of a 1:1 solution into the HSA solution, thoroughly mixing them to establish phenolic acid to HSA molar ratios of 0, 0.74, 1.48, 2.22, 2.96, and 3.7. The measurement stipulations were as follows: the excitation wavelength of HSA was 280 nm, the slit width was set to 15 × 15 nm, the emission spectrum scan range is 300–500 nm, and the fluorescence spectral changes of the HSA solution were determined.

To negate the inner filter effect of fluorescence, the following formula was applied to correct the fluorescence intensity:

$$F_{\text{cor}}=F_{\text{obs}}\cdot e^{\left(A_{1}+A_{2}\right)/2}$$

In the formula, *F*_cor_ symbolizes the fluorescence intensity after correction of the emission wavelength, *F*_obs_ represents the fluorescence intensity prior to the correction of the emission wavelength, A_1_ and A_2_ were respectively the absorbance values of the monomer at the emission and emission wavelength locations.

#### 2.4.6 Competitive binding [32]

2 mL of HSA solution was transferred into a centrifuge tube and separately introduce CA and FA solutions, establishing a molar ratio of CA and FA to HSA of 1:1, thoroughly shaken and allowed to react for 20 min. Subsequently, five batches of 5 μL of warfarin solution and ibuprofen solution (both at a concentration of 1.52 × 10^−4^ mol L^−1^) were added as site fluorescence probes. The measurement parameters were as follows: excitation wavelength was 280 nm, the slit width was set at 15 × 15 nm, the emission spectrum scan range was 300–500 nm, and the fluorescence spectral changes of the phenolic acid-HSA system was determined.

#### 2.4.7 Synchronous fluorescence spectroscopy [33]

2 mL of HSA solution was transferred into a 1 cm quartz cuvette, sequentially introduce five batches of 5 μL CA solution, FA solution, thoroughly mixed them to establish phenolic acid to HSA molar ratios of 0, 0.74, 1.48, 2.22, 2.96, and 3.7. Allow the reaction to fully complete under stationary conditions. Setting a fixed wavelength difference of Δλ = 15 nm and Δλ = 60 nm with a slit width of 15 × 15 nm, using an excitation wavelength of 280 nm, and conducting a synchronous fluorescence spectral scan in the range of 300–500 nm.

#### 2.4.8 Circular dichroism spectrum [22,34]

2 mL of HSA solution was transferred into a 1 cm quartz cuvette, sequentially introducing appropriate amounts of CA, FA, and a 1:1 solution, and measuring the changes in the circular dichroism (CD) spectrum of the HSA solution. The measurement parameters were as follows: a scanning wavelength range of 200–400 nm, a spectral bandwidth of 1 nm, and a 0.50 mol L^−1^ Tris-HCl buffer solution (pH = 7.4). The experiment was conducted under N_2_ conditions. The conformation was calculated online by the BeStSel software.

#### 2.4.9 Molecular docking [35]

The molecular docking process was accomplished using the software package from Accelrys' Discovery Studio 2019 (DS) by Accelrys Inc. The HSA structure was sourced from the PDB database (pdb code:1H9Z), where water molecules, heteroatoms, and multiple conformations of amino acid residues in the HSA crystal structure were removed. The phenolic acid were in the deprotonated form. Docking was performed under the CHARMm force field by the software package's Flexible Docking module. Solvation calculations were performed on the complex, and compensatory ions Na^+^ and Cl^−^ were added to the entire system to approximate the conditions within the human body. Finally, minimization calculations were performed to determine the most probable conformation of the complex.

#### 2.4.10 Molecular dynamics (MD) simulation [22,36]

The molecular docking results were subjected to a MD simulation of 100 ns duration in GROMACS 2019. The CHARMM36 all-atom force field was applied to the solution, and the force field parameters for deprotonated phenolic acids were obtained from the CGenFF server. The TIP3P water model was selected as the solvent, enveloping the system with a 7 Å layer of water molecules. The charge of the simulation system was balanced by adding Na^+^ and Cl^−^. Within the simulation system, the Particle Mesh Ewald (PME) method was employed to calculate long-range electrostatic interactions. Energy optimization of the entire system was carried out using the steepest descent and conjugate gradient methods. A time step of 2 fs was used for all simulations. NVT equilibration was performed with V-rescale thermostat at 303 K and constant volume for 200 ps Then NPT equilibration was done with the scheme of Parrinello-Rahman barostat at 1.0 bar and 303 K for 200 ps. Finally, 100 ns production simulation was executed at 303 K with NPT scheme of Parrinello-Rahman barostat at 1.0 bar. After the MD simulation, periodic boundary conditions were removed from the system. MD trajectory analysis was performed using VMD 1.9.3a to yield a series of parameters.

#### 2.4.11 Quantum chemical calculations [37]

The quantum chemical calculations of the phenolic acid in the deprotonated form are performed using Gaussian 16 and GaussView 6.0. The Gaussian computations employ the density functional theory (DFT) under the B3LYP ground state, setting the basis set as 6-311G*. The molecular HOMO-LUMO orbitals are plotted and their energy levels are calculated.

## 3 Results

### 3.1 Screening for SR hemostatic efficacy proteins

(1) Construction of a hemostatic target network for SR phenolic acids

**Fig. 2:**
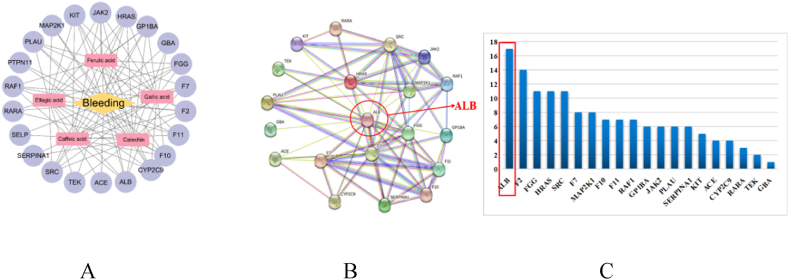
Result of network pharmacology. A. SR phenolic acid hemostatic target network diagram, B. Construction of the PPI network, C. The scoring bar of core targets.

A total of 356 targets for SR phenolic acids were identified through the Pharm Mapper database, and gene targets associated with hemorrhage were collected from the GeneCards database, yielding 132 targets. Twenty-two intersecting targets between the monomer and hemorrhage were obtained using R language. Detailed information about the potential targets can be found in Table 1. The information on the potential hemostatic action targets of phenolic acids was imported into Cytoscape software to construct a hemorrhagic-SR phenolic acid-target Network diagram, as shown in Fig. 2A. In the figure, the pink rectangular nodes represent the phenolic acid monomers, the purple circular nodes represent the action targets, and the orange diamond nodes represent the disease. The lines connecting them represent the interactions between the monomer, the target protein, and the disease. Twenty-two potential hemostatic targets for the effective hemostatic components of SR phenolic compounds were identified.

(2) Screening of the PPI network and identification of core targets

**Table 1:** Potential target information of tannin monomer for hemostasis.

Compound	Targets
CA,FA,GA,EA,C	SRC, F2, ALB, F10, CYP2C9, PLAU, JAK2, F11, TEK, MAP2K1, KIT, RARA, HRAS, GP1BA, FGG, RAF1, ACE,GBA, SERPINA1, F7, PTPN11, SELP

A protein-protein interaction (PPI) network was constructed utilizing the STRING online server (Fig. 2B). Each node represents a target protein, with each edge signifying the relationship between targets. The nodes situated at the center and with a higher number of edges were more likely to be core targets. Albumin (ALB) was located in the central region of the network, connecting numerous nodes and thus occupying a pivotal position within the network. The CytoHubba plugin was utilized to screen the core targets within the PPI network. The topological parameters of degree, closeness, and betweenness in the PPI network were calculated [23], and twenty targets were screened out based on these three topological parameters. The degree value reflects the significance of the interaction between the targets. ALB has the highest degree value, indicating its crucial associative role within the PPI network and suggesting that it might be the primary target for the hemostatic effect of the active components in SR (Fig. 2C). The Uniprot database showed that the protein corresponding to ALB was serum albumin. Hence, HSA was selected for further investigations.

### 3.2 SDS-PAGE

**Fig. 3:**
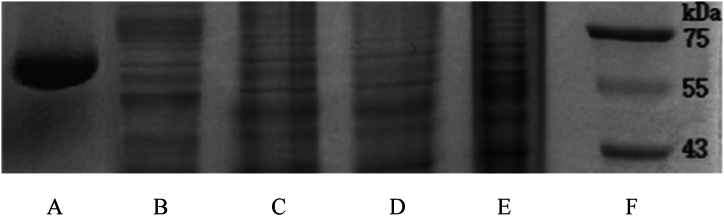
Phenolic acid and HSA electrophoretogram. A. HSA blank group, B. plasma blank group, C. CA + plasma, D. FA + plasma, E. 1:1+plasma, F. Marker.

SDS-PAGE leverages the differential molecular weight changes resulting from the interaction between drugs and proteins, which manifest as alterations in the protein migration bands, to glean information on the binding of small molecules to biological macromolecules. The experimental results are depicted in Fig. 3. The position of the HSA band in the untreated plasma was determined using a molecular weight of 67 kDa for HSA, and referring to the marker (Fig. 3F) and the blank HSA band (Fig. 3A). As illustrated in Fig. 3B to E, upon the addition of phenolic acid, the position of the HSA band shifted backward, indicating that CA and FA bound with HSA in the plasma, thereby slowing its migration rate through the gel. This suggests that HSA is one of the sites of action for these drugs in plasma. The stronger the binding, the slower the migration. The order of the band shift was 1:1 > FA > CA, signifying that the mixture in plasma bound more strongly to HSA than the individual components, with FA binding more strongly than CA.

### 3.3 UV–Vis absorption spectroscopy

**Fig. 4:**
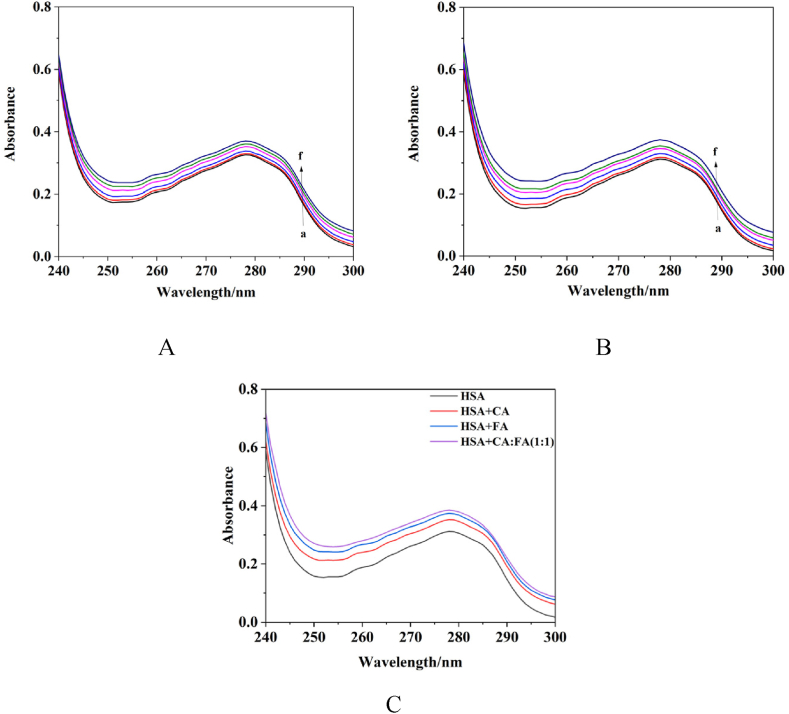
UV–Vis absorption spectrum. A. HSA + CA, B. HSA + FA, C. HSA + phenolic acid (a:0 μL, b:5 μL, c:10 μL, d:15 μL, e:20 μL, f:25 μL).

Ultraviolet spectroscopy can be employed to study whether a complex forms between proteins and small molecules based on the changes in absorption peaks. The ultraviolet–visible spectroscopy results revealed that there was a gradual increase in the intensity of the characteristic absorption peak of HSA at 278 nm with the addition of phenolic acid, and a blue shift in the peak position, indicating that the peptide chains of HSA were extending (Fig. 4). This exposes tryptophan and amino acid residues, resulting in a hyperchromic effect. The phenolic acid induces the exposure of the aromatic heterocyclic hydrophobic groups, such as those of tryptophan and tyrosine residues, which are typically enveloped within the HSA molecule, leading to a blue shift in the absorption peak. The greater the increase in peak intensity and the shift in peak position, the stronger the interaction of phenolic acid with HSA.

A comprehensive analysis of the results shown in Fig. 4 and Table 2 revealed that SR phenolic acids interacted with HSA, with a binding strength in the following order: 1:1> FA > CA.

**Table 2:** Changes in the ultraviolet absorption intensity following the addition of phenolic acid to the HSA solution.

Group	Δ(%)^a^
HSA + CA	12.82
HSA + FA	19.87
HSA+1:1	24.68

a Δ(%)=(*A*-*A*_0_)/*A*_0_ × 100 % (represents the ultraviolet absorption intensity of the HSA solution prior to the addition of the drug, while *A* denotes the ultraviolet absorption intensity of the HSA solution after the drug has been introduced).

### 3.4 Fluorescence quenching

**Fig. 5:**
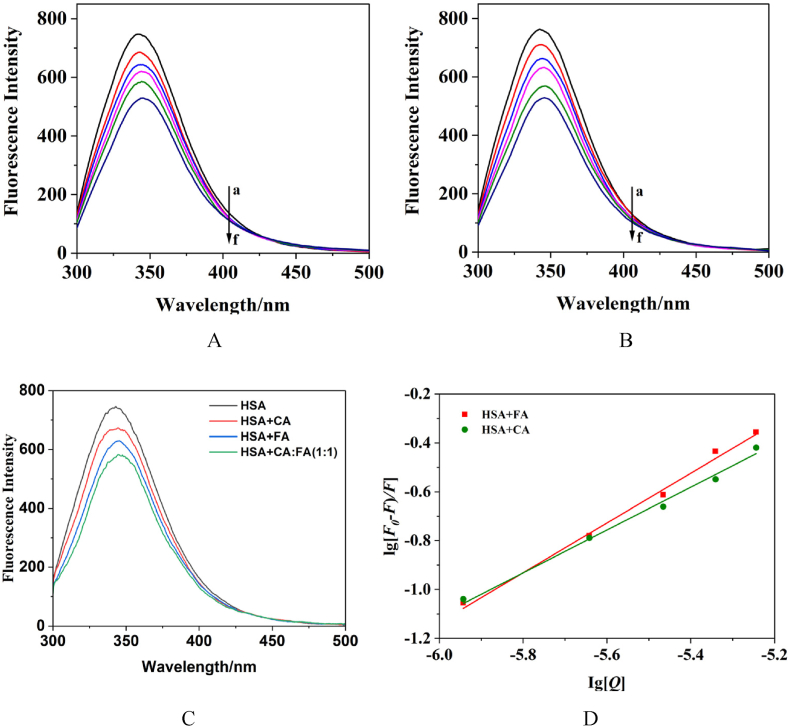
Fluorescence quenching of the interaction between phenolic acid and HSA. A. HSA + CA, B. HSA + FA, C. HSA + phenolic acid, D. Double logarithmic regression curve (a:0 μL, b:5 μL, c:10 μL, d:15 μL, e:20 μL, f:25 μL).

The fluorescence of HSA originates from its aromatic amino acid residues (predominantly Tyr and Trp). The interaction between small molecules and HSA was studied using fluorescence quenching methods (Fig. 5). As the concentration of the phenolic acid solution increased, there was a notable decrease in the fluorescence peak intensity of HSA at 340 nm, indicating quenching of its intrinsic fluorescence. This suggests that phenolic acid forms a complex with HSA, altering the conformation of HSA.

The fluorescence quenching effect can be utilized to determine the binding constant and the number of binding sites for the interaction. The formula is as follows: $\mathrm{lg}\frac{F_{0}-F}{F}=\mathrm{lg}\;K_{a}+\text{nlg}\left[Q\right]$. In this equation, *F*_0_ represents the fluorescence intensity of the HSA system prior to adding the sample, *F* is the fluorescence intensity after the sample is added, n is the number of binding sites, *K*_a_ is the binding constant, and [*Q*] is the concentration of the sample [30]. The plot of $\mathrm{lg}\frac{F_{0}-F}{F}$ against lg[Q], where the intercept represents *K*_a_, and the slope corresponds to n, is presented in Fig. 5D. For CA + HSA, *K*_a_ was 4.15 × 10^4^ L mol^−1^ with n = 0.91; for FA + HSA, *K*_a_ was 4.97 × 10^4^ L mol^−1^, with n = 1.02. This indicates that FA could bind more strongly than CA, with both FA and CA binding to a single site on HSA. A comprehensive analysis of the results in Fig. 5 and Table 3 revealed that the 1:1 exhibited the strongest quenching effect on HSA, and the binding strength of the phenolic acid system with HSA followed the order 1:1 > FA > CA.

**Table 3:** The changes in fluorescence intensity of the interaction between phenolic acid and HSA.

Group	Δ(%)^a^
HSA + CA	−9.78
HSA + FA	−15.56
HSA+1:1	−21.92

a Δ(%)=(*F*-*F*_0_)/*F*_0_ × 100 % (*F*_0_ represents the fluorescence intensity of the HSA solution before the introduction of the drug, while *F* denotes the fluorescence intensity of the HSA solution after the drug has been added).

### 3.5 Competitive binding

Competitive binding experiments were conducted to ascertain the binding location of SR phenolic acid with HSA. HSA possesses four binding sites, with the Suldow site I and site II serving as the principal binding sites for drug molecules. Warfarin and ibuprofen, which specifically bind to known sites on HSA, were used as site markers in fluorescence probes for the competitive binding experiments. Warfarin binds to the Sudlow site I while ibuprofen binds to the Sudlow site II, allowing for the respective monitoring of HSA's Sudlow site I and site II [32].

The percentage of competition between the drug and the binding site marker was calculated according to the formula $\text{Displacement}=\frac{F_{2}}{F_{1}}\times 100\%$. In the formula, *F*_1_ is the fluorescence intensity of HSA + phenolic acid in the absence of a binding site marker, and *F*_2_ is the fluorescence intensity of HSA + phenolic acid in the presence of a binding site marker. *F*_2_/*F*_1_ was plotted against the ratio of the concentration of the binding site marker to the concentration of the protein.

**Fig. 6:**
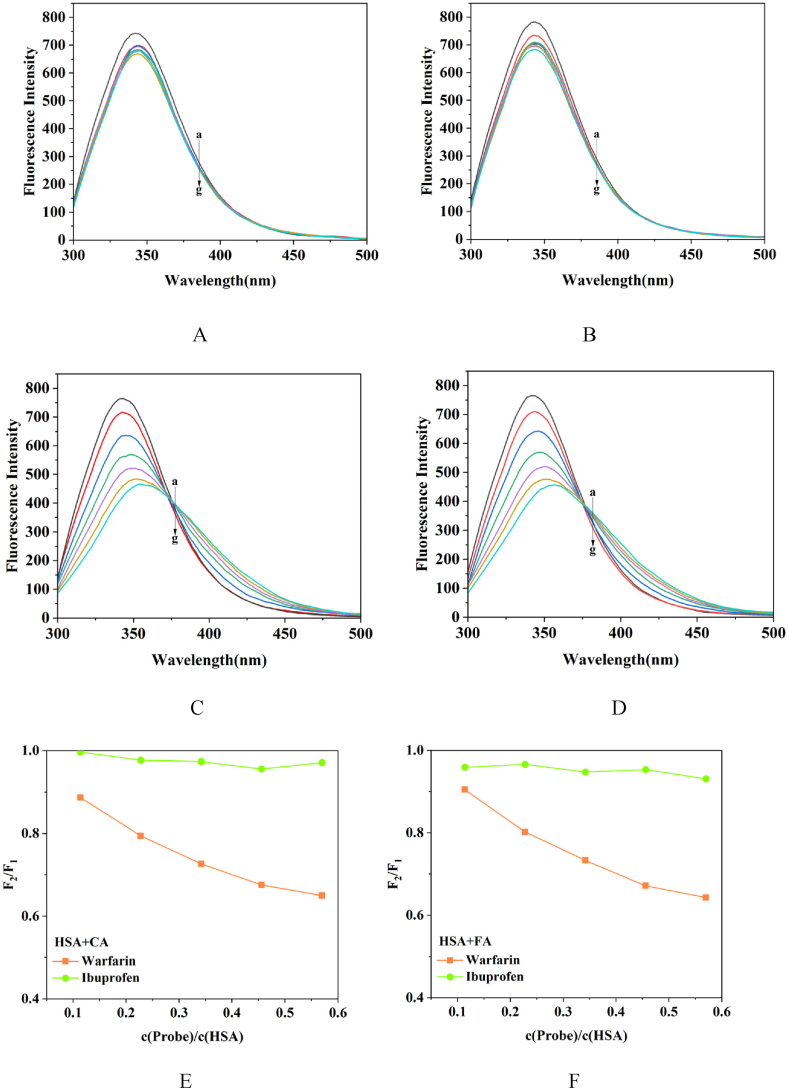
Competitive binding. A. HSA + CA + ibuprofen, B. HSA + FA + ibuprofen, C. HSA + CA + warfarin, D. HSA + FA + warfarin, E. CA system competition percentage, F. FA system competition percentage (a. HSA, b. HSA + phenolic acid, c–g: 5 μL, 10 μL, 15 μL, 20 μL and 25 μL of marker was added).

As depicted in Fig. 6, with the increase in warfarin concentration, a decrease in the fluorescence intensity of the interaction between HSA and phenolic acid was observed. No significant change in the fluorescence intensity of the system occurred upon the addition of ibuprofen. This indicated that both CA and FA compete for binding with warfarin, primarily occupying the Sudlow site I (subdomain IIA) of HSA.

### 3.6 Synchronous fluorescence

**Fig. 7:**
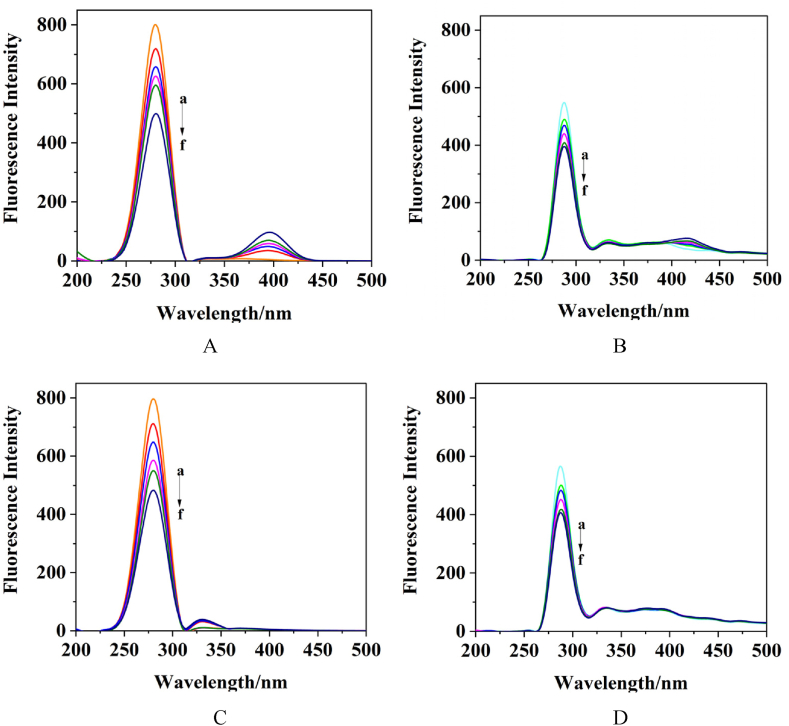
Synchronous fluorescence spectrum of the interaction. A. HSA + CA, Δ*λ* = 60 nm, B.HSA + CA, Δ*λ* = 15 nm, C. HSA + FA, Δ*λ* = 60 nm, D. HSA + FA, Δ*λ* = 60 nm (a:0 μL, b:5 μL, c:10 μL, d:15 μL, e:20 μL, f:25 μL).

The effect of CA and FA on the structure of HSA was revealed using synchronous fluorescence spectroscopy. The fluorescence characteristics of tyrosine and tryptophan residues were analyzed by scanning fluorescence spectra with fixed wavelength differences (Δλ) of 15 nm and 60 nm. The synchronous fluorescence obtained at a Δλ of 15 nm provides information on the tyrosine residues within HSA, while at a Δλ of 60 nm, it yields insights into the tryptophan residues [33]. Increasing the concentration of the phenolic acid solution at a constant HSA concentration reduced the fluorescence intensity for both tyrosine and tryptophan in the synchronous fluorescence spectra (Fig. 7). This decrease in fluorescence intensity at a Δ*λ* of 15 nm and a Δ*λ* of 60 nm with increasing phenolic acid concentrations suggested an interaction between CA, FA, and HSA, leading to the formation of complexes and subsequent fluorescence quenching.

The quenching percentage of the system's synchronous fluorescence was calculated according to the formula: $R_{SFQ}=1-F/F_{0}$, where *F*_0_ and *F* represent the synchronous fluorescence intensities before and after adding small molecule solutions, respectively. At Δ*λ* = 60 nm, the *R*_*SFQ1*_ for FA was 58.1 %, and for CA, it was 46.85 %. At Δ*λ* = 15 nm, the *R*_*SFQ2*_ for FA was 43.14 %, and for CA, it was 32.98 %. The *R*_*SFQ1*_ for both interactions with HSA was greater than *R*_*SFQ2*_, and the fluorescence quenching intensity at Δ*λ* = 60 nm was significantly higher than at Δ*λ* = 15 nm. This indicated that the binding regions of phenolic acids with HSA were concentrated near Trp214, the only one tryptophan residue in HSA, and that the binding strength for FA was greater than for CA.

### 3.7 Circular dichroism spectroscopy

**Fig. 8:**
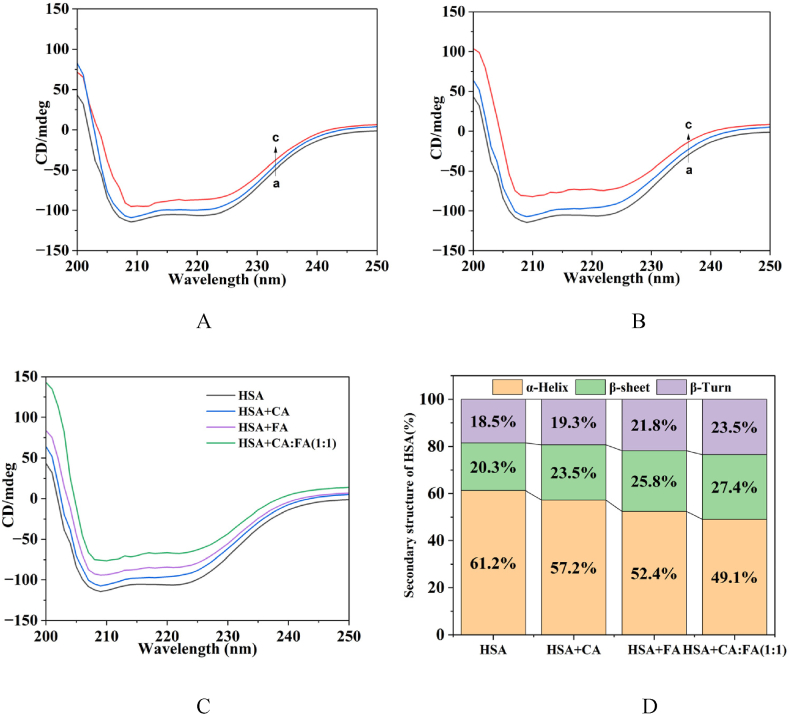
CD spectroscopy. A. HSA + CA, B. HSA + FA, C. HSA + phenolic acid, D. changes of the secondary structural (a:0 μL, b:25 μL, c:50 μL).

The secondary structural changes in HSA were monitored using circular dichroism (CD) spectroscopy, and the alterations in the secondary structure conformations were quantified through online analysis using BeStSel software (Fig. 8). HSA exhibited two negative absorption peaks in the CD spectrum at 208 and 222 nm, characteristic of the α-helix structure. The intensity of the two negative absorption peaks of HSA decreased with the addition of phenolic acids. However, the shape and position of the shoulder peaks remained largely unchanged, indicating that the incorporation of small molecules merely reduced the content of the α-helix content, which still maintained their dominance. There was a decrease in the proportion of α-helices following the addition of phenolic acids, accompanied by an increase in the content of β-turns and β-sheets. The transformation from α-helices to β-turns and β-sheets led to changes in the hydrophobic environment surrounding the amino acid residues. The extent of conformational changes followed the order: 1:1 > FA > CA, consistent with the SDS-PAGE, UV–Vis, and fluorescence spectroscopy results.

### 3.8 Molecular docking

Molecular docking vividly illustrates the binding interactions between drugs and proteins, aiding in the theoretical determination of the mechanisms and modes of drug-protein interactions, thereby complementing the conclusions drawn from experimental results.

Fluorescence spectroscopy indicated that the binding ratio of phenolic acids to HSA is approximately 1:1. Therefore, one molecule of phenolic acid was docked with one molecule of HSA for molecular docking. The deprotonated phenolic acid molecule was defined as the ligand, and flexible docking was performed using Discovery Studio. UV, fluorescence, and CD spectroscopy simulated reactions under physiological conditions. The Tris-HCl buffer solution contained 0.1 mol L^−1^ NaCl to maintain ionic strength. Consequently, the solvation calculations in the simulation also incorporated compensations for Na^+^ and Cl^−^.

**Fig. 9:**
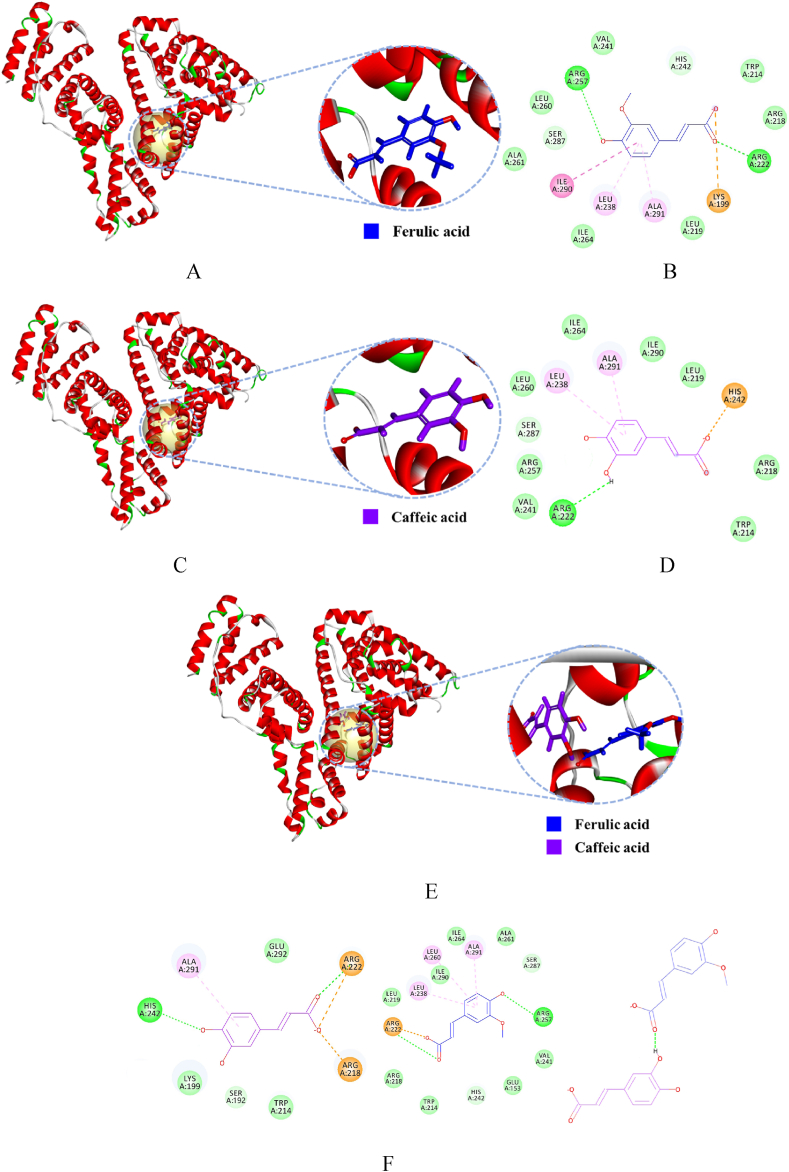

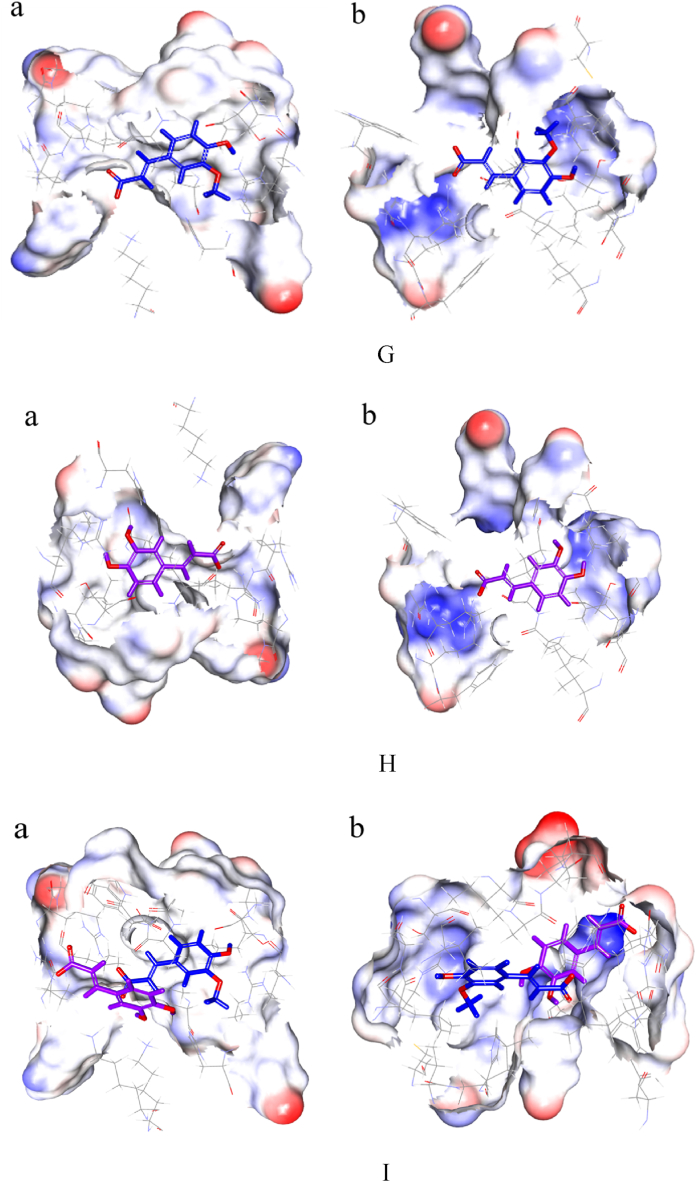
Molecular docking. A. Interaction between FA and HSA (FA displayed in sticky). B. 2D diagram of the interaction between FA and HSA (green dashed lines represent hydrogen bonds, purple dashed lines denote hydrophobic interactions, and yellow dashed lines denote salt bridge). C. Interaction between CA and HSA (CA displayed in sticky). D. 2D diagram of the interaction between CA and HSA (green dashed lines represent hydrogen bonds, purple dashed lines denote hydrophobic interactions, and yellow dashed lines denote salt bridge). E. Interaction between 1:1 and HSA (FA displayed in blue sticky and CA in purple). F. 2D diagram of the interaction between 1:1 and HSA (green dashed lines represent hydrogen bonds, purple dashed lines denote hydrophobic interactions, and yellow dashed lines denote salt bridge, FA displayed in blue sticky and CA in purple). G. Interface of the active pocket where FA and HSA interace (FA displayed in sticky, a is hydrophobic interface, b is hydrophilic interface). H. Interface of the active pocket where CA and HSA interace (CA displayed in sticky, a is hydrophobic interface, b is hydrophilic interface). I. Interface of the active pocket where 1:1 and HSA interace (FA displayed in blue sticky and CA in purple, a is hydrophobic interface,b is hydrophilic interface).

The binding pockets of CA with HSA included amino acids Trp214, Arg218, Leu219, Arg222, Leu238, Val241, His242, Arg257, Leu260, Ile264, Ser287, Ile290 and Ala291. In contrast, the binding pocket for FA has two more residues: Lys199 and Ala261. Whereas the 1:1 binding of phenolic acids with HSA included amino acids Glu153, Ser192, Lys199, Trp214, Arg218, Leu219, Arg222, Leu238, Val241, His242, Arg257, Leu260, Ala261, Ile264, Ser287, Ile290, Ala291, and Glu292 (Fig. 9A–F). These residues were all located within the Sudlow site I of subdomain IIA, consistent with the experimental findings in 3.5 competitive binding.

Upon entering the active pocket of HSA, phenolic acids engaged in hydrogen bonding, hydrophobic, and hydrophilic interactions with the surrounding amino acids. In the interaction analysis, FA formed three hydrogen bonds with HSA, CA formed two hydrogen bonds, and the 1:1 formed seven hydrogen bonds, with CA and FA additionally forming two hydrogen bonds between themselves (Fig. 9B,D, and 9F). Detailed information is presented in Table 4.

**Table 4:** The hydrogen bond information formed by phenolic acid and HSA.

Compound	X—H…Y	d (X—H)	d (H…Y)	d (X … Y)	∠XHY
FA	Arg222:NH2-HH21 … O-C9(FA)	0.99	2.33	3.32	117.25
FA	Arg257:NH1-HH12 … O-C4(FA)	1.34	2.53	3.21	123.53
CA	Arg222: NE-HE … O-C3(CA)	1.01	2.29	3.11	137.15
1:1	Arg222:NH1-HH12 … O-C9(FA)	1.12	2.84	3.41	119.53
1:1	Arg257:NH2-HH22 … O-C4(FA)	1.46	2.71	3.13	150.72
1:1	Arg222:NE-HE … O-C9(CA)	1.25	2.37	3.18	136.94
1:1	His242:NH2-HH22 … O-C4(CA)	1.85	1.99	2.93	154.92
1:1	CA:C3-O-H...O-C9(FA)	0.95	2.09	2.97	151.12

Fig. 9G and H depicted the interfaces of hydrophobic and hydrophilic amino acid residues. Calculations revealed that the interaction energy between the hydrophobic amino acid residues and the 1:1, FA, and CA were −214.5 kJ/mol, −121 kJ/mol, and −51.4 kJ/mol, respectively, while the interaction energy with hydrophilic amino acid residues was −82.1 kJ/mol, −38.1 kJ/mol, and −17.9 kJ/mol, respectively. The interface diagrams indicated that the hydrophobic interactions between phenolic acids and the active pocket's amino acids were more pronounced than hydrophilic interactions.

Calculations determined that the electrostatic energy between FA and HSA was −242.5 kJ/mol, while the van der Waals energy was 27.5 kJ/mol. The electrostatic energy between CA and HSA was 27.81 kJ/mol, with a van der Waals energy of −197.8 kJ/mol. For the 1:1 with HSA, the electrostatic energy was −257.9 kJ/mol and the van der Waals energy was 26.8 kJ/mol. The electrostatic energy in CA system was lower than the van der Waals energy in the FA and 1:1 system, indicating that the interaction with HSA was primarily electrostatic. Conversely, the van der Waals energy was lower than the electrostatic energy for CA, suggesting that the predominant interaction with HSA was through intermolecular forces.

The formula for binding energy calculation was: $E_{binding}=E_{complex}-\;E_{ligand}-\;E_{receptor}$, where $E_{binding}$ represents the binding energy between the small molecule and HSA, $E_{complex}$ denotes the energy of the formed complex post-docking, $E_{Ligand}$ and $E_{receptor}$ represent the energies of the small molecule and HSA, respectively. The calculated △*G* between the 1:1 complex and HSA was −185.2 kJ/mol. The binding energy △*G* between FA and HSA was −142.7 kJ/mol, and between CA and HSA, the △*G* was −107.3 kJ/mol. It was evident from the △*G* values that the stability of the binding between phenolic acids and HSA was in the order of 1:1 > FA > CA. This result was consistent with the order determined by SDS-PAGE and the spectroscopic experiments.

The variation in binding strength among the three could be attributed to the following factors: First, within the mixture, FA and CA bound to different regions within the Sudlow site I, which expanded the binding active pocket and increased the number of hydrogen bonds formed, leading to a synergistic effect that resulted in stronger binding than that of the individual components. The number and strength of hydrogen bonds formed by FA were greater than those formed by CA. Second, the interactions between the mixture, FA, and HSA were electrostatic, whereas the interactions between CA and HSA were intermolecular. The electrostatic forces were stronger than the van der Waals forces. Third, the structural differences between FA and CA, with FA being less polar than CA, made it easier for FA to insert into the hydrophobic environment of the surrounding amino acids.

### 3.9 MD simulation

**Fig. 10:**
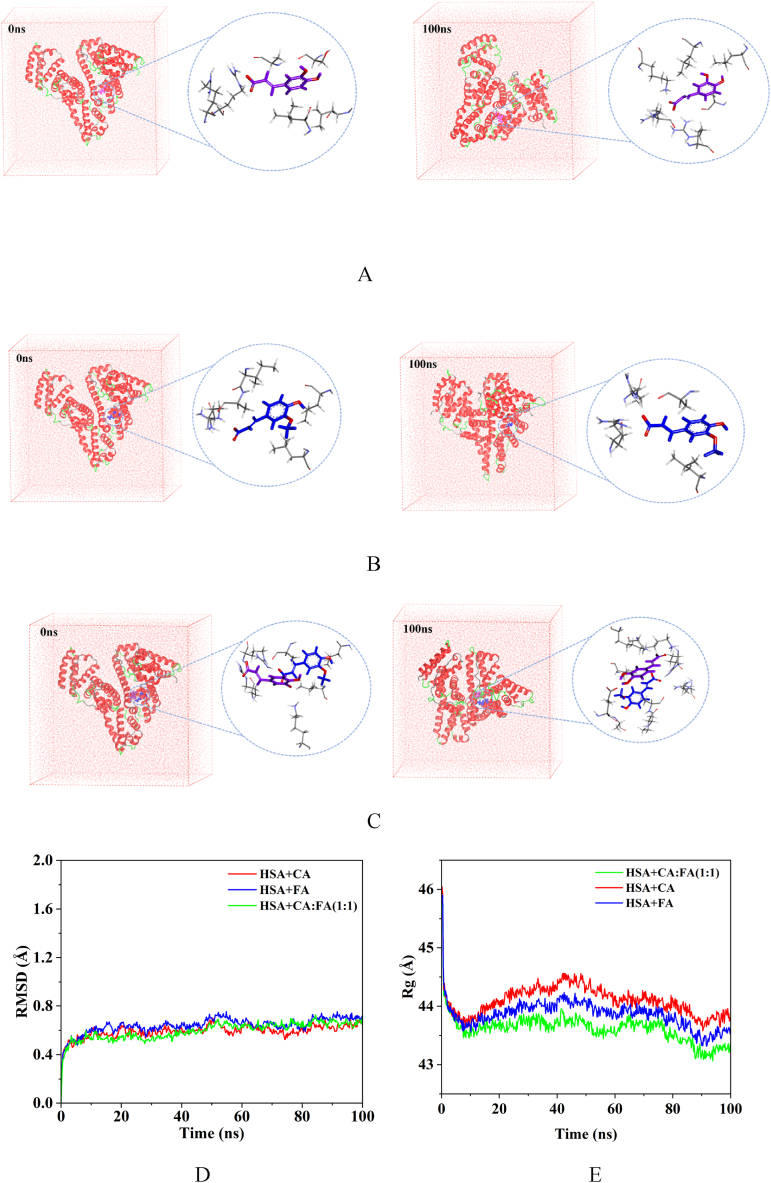

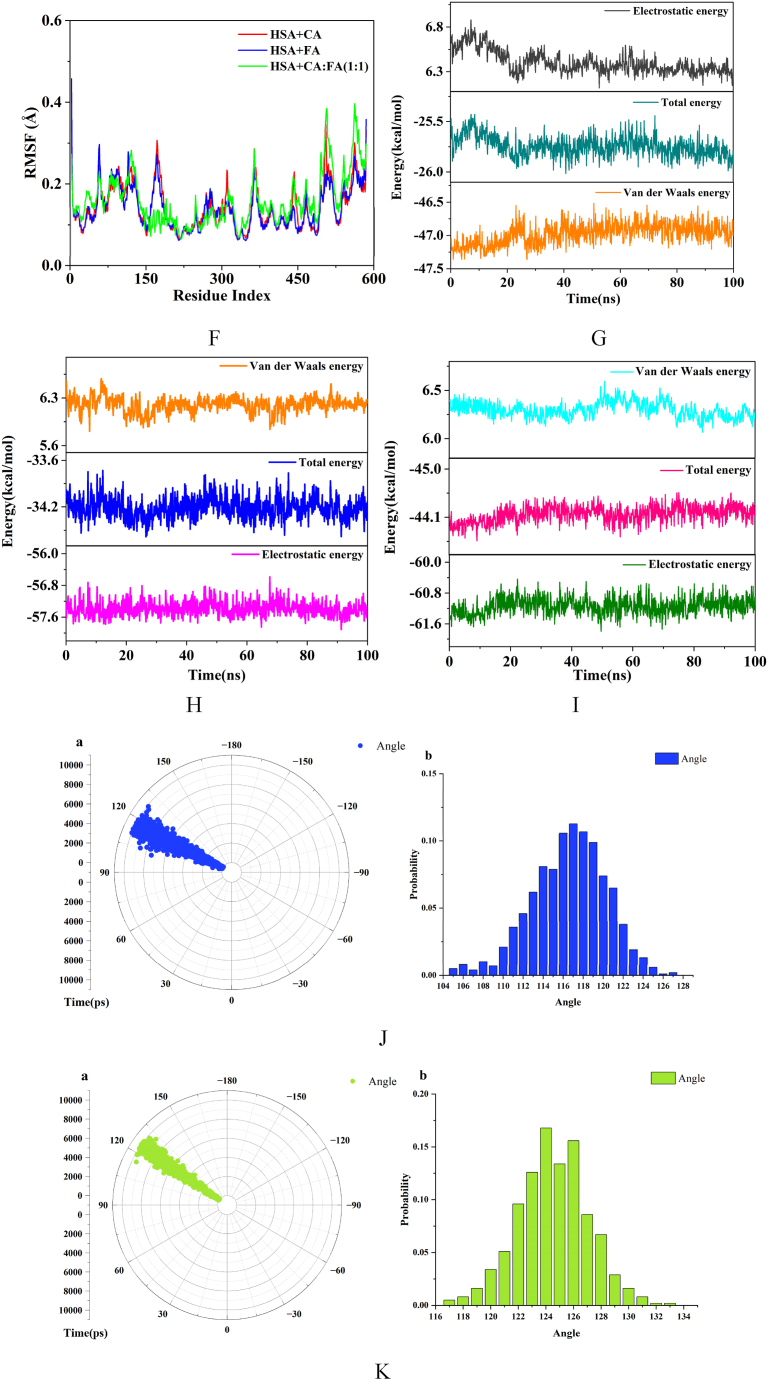

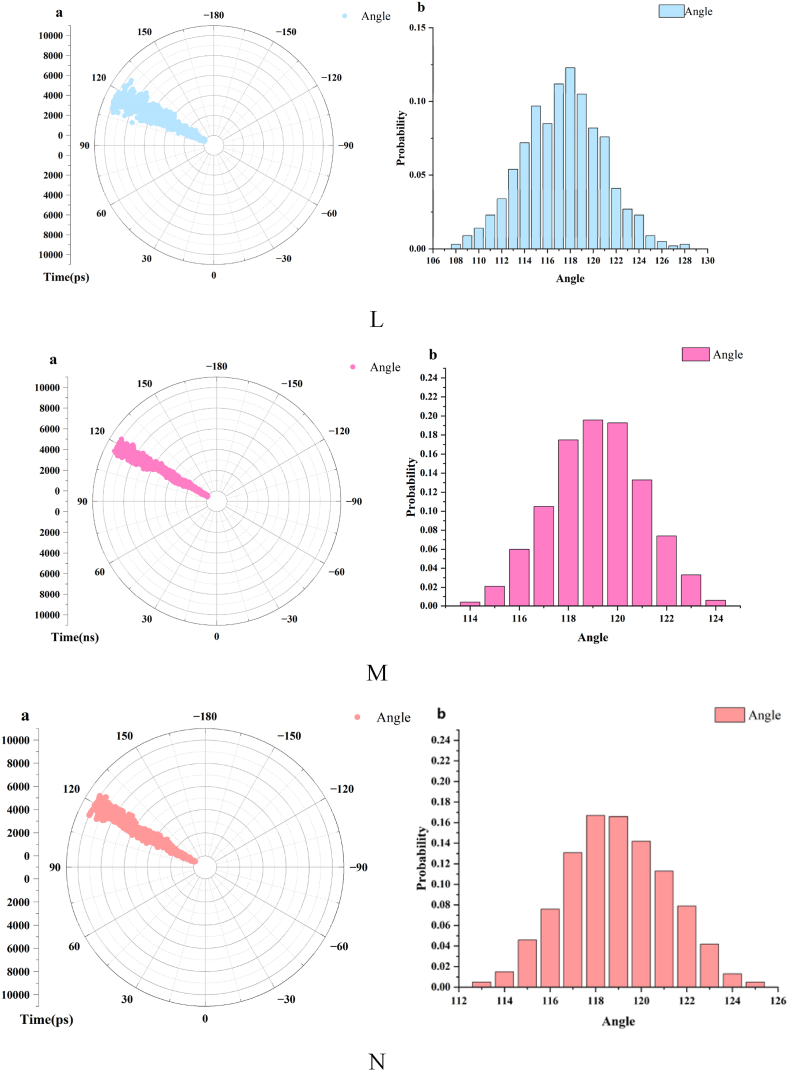
Results of MD. A. CA + HSA binding diagram (0 ns, 100 ns), B. FA + HSA binding diagram (0 ns, 100 ns). C.1:1 + HSA binding diagram (0 ns, 100 ns), D. Analysis of RMSD, E. Analysis of Rg. F. Analysis of RMSF, G-I. Analysis of energy (HSA + CA, HSA + FA, HSA+1:1). J. Variation of rotatable bond angle of FA∠C3-O1-C10 (a:Time-Angle radial plot, b:Torsional range of rotatable bond angles). K. Variation of rotatable bond angle of FA∠O3-C9-O4 (a:Time-Angle radial plot, b:Torsional range of rotatable bond angles). L. Variation of rotatable bond angle of FA∠O4-C9-C8(a:Time-Angle radial plot,b:Torsional range of rotatable bond angles). M. Variation of rotatable bond angle of CA∠O4-C9-C8 (a:Time-Angle radial plot, b:Torsional range of rotatable bond angles). N. Variation of rotatable bond angle of CA∠O3-C9-C8 (a:Time-Angle radial plot, b:Torsional range of rotatable bond angles).

The results of the molecular docking of phenolic acid with HSA were subjected to a 100 ns MD simulation using GROMACS 2019 to dynamically analyze their interactions. Overall stability was visually examined through metrics such as root mean square deviation (RMSD), radius of gyration (Rg), root mean square fluctuation (RMSF), energy values, and variations in rotatable bond angles. The findings are presented in Fig. 10.

Fig. 10A–C illustrate the binding of CA, FA, and 1:1 with HSA at 0 ns and 100 ns, respectively. The binding of phenolic acid with HSA remained stable, with the small molecules still bounded to site I of HSA after 100 ns, showing no signs of molecular escape. The small molecules exhibited a certain degree of deviation during the simulation, with the degree of flipping of FA being greater than that of CA.

The stability of the protein system was characterized by calculating the average deviation (RMSD) between the original conformation and the protein conformation during simulation. After binding with HSA, the RMSD values of both steadily converged towards equilibrium after 10 ns, with no further significant fluctuations, indicating that the simulated system had reached a state of binding equilibrium (Fig. 10D).

Rg represents the maximum distance of atoms from the centroid. It reflects the system's stability and the structural changes in HSA post-binding through the magnitude of fluctuations. An increase in the Rg value indicates protein structure expansion, while a decrease suggests protein structure compaction. The Rg plots for the complexes of CA, FA, and 1:1 with HSA are shown in Fig. 10E. There was a noticeable contraction in the HSA structure at the initial stages of binding with phenolic acid, which then stabilized after 10 ns. The Rg variation in the HSA + CA system was smaller than that in the HSA + FA system over 100 ns, indicating that the structural alteration of HSA by CA was less than that by FA, with the alterations being most significant in 1:1.

The root mean square fluctuation (RMSF) indicates changes in the flexibility of the protein after binding. The fluctuations within the amino acid residue range of 200–300 were minimal for both the CA and FA systems, suggesting stable binding of CA and FA with HSA in this region (Fig. 10F). The range of stable fluctuations extended to 150–300 after binding with the 1:1, indicating an even more stable binding of the mixture. The range of stable fluctuations encompassed the amino acid residues at site I of HSA.

The dynamic analysis of the interaction energies within the system during the simulation process is presented in Fig. 10G–I. After CA bound with HSA, the van der Waals energy was lower than the electrostatic energy, whereas the electrostatic energy was lower than the van der Waals energy for FA's binding with HSA. This suggests that the binding of FA with HSA was predominantly governed by electrostatic interactions, whereas CA binds with HSA through intermolecular forces. The electrostatic energy was lower than the van der Waals energy upon binding with 1:1, indicating that the mixture bound with HSA primarily through electrostatic interactions.

The aforementioned results are consistent with those obtained from fluorescence binding site experiments and molecular docking studies. This consistency suggested that the molecular docking outcomes were possessed of commendable dynamic stability and the result from 3.7 molecular docking were reliable.

Changes in the torsion angles can reflect the structural alterations of phenolic acids upon binding with HSA. By calculating the variation in the rotatable bonds of phenolic acids over 100 ns, we can analyze their stability and structural changes during binding. Greater changes in angle suggested that the small molecule became more extended and its side chains were more elongated after binding. The rotatable angles of the phenolic acids were sourced from the PubChem database, with PubChem CID for CA of 689043, and 445858 for FA. The rotatable angles for FA were ∠C3-O1-C10, ∠O3-C9-O4, and ∠O4-C9-C8, and for CA were ∠O4-C9-C8 and ∠O3-C9-C8. The Gromacs angle module was used to calculate the time-dependent changes in these rotatable angles, as shown in Fig. 10J–N, a is a time-angle radial plot that indicates the angles of the rotatable bonds, and b is the range of torsion for the rotatable bonds. Both FA and CA have rotatable angles around 120°, with the range and variation detailed in the graphs and Table 5. According to Table 5, FA has three rotatable angles and CA has two, with their average changes in torsion angles of 19°and 11°, respectively. FA has more rotatable angles and a greater degree of change than CA, indicating that FA possesses better structural flexibility and is more prone to twisting. When bound to HSA, FA underwent greater structural alteration and its side chains became more extended. This was related to the structural differences between FA and CA; both molecules have a similar phenyl ring-conjugated structure. However, FA has an -OCH_3_ at C3 while CA has an -OH, making CA more hydrophilic than FA. Consequently, FA's side chains can more readily extend and insert into the hydrophobic environment of HSA, resulting in a stronger binding. The molecular docking results indicated that the binding stability of FA with HSA was superior to that of CA, suggesting that the greater the flexibility of a small molecule, the more stable its binding.

**Table 5:** The torsional angle variations of phenolic acids.

Compound	Bond angle	Range of variation	Δ	Average variation
FA	∠C3-O1-C10	105°–127°	22°	19°
FA	∠O3-C9-O4	117°–133°	16°	
FA	∠O4-C9-C8	108°–128°	20°	
CA	∠O4-C9-C8	114°–124°	10°	11°
CA	∠O3-C9-C8	113°–125°	12°	

### 3.10 Quantum orbital analysis

**Fig. 11:**
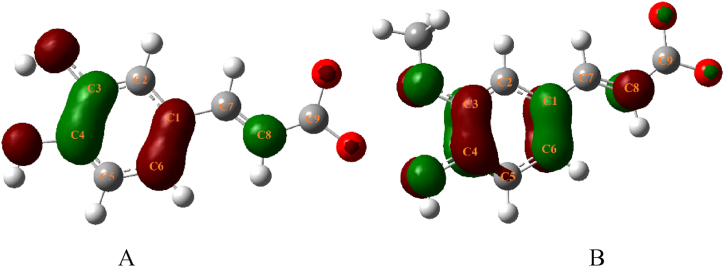
Orbital distribution for HOMO and LUMO. A. FA, B. CA.

Quantum chemical computations could delineate the atomic bonding properties during the binding process, and the calculation of the highest occupied molecular orbital (HOMO) and the lowest unoccupied molecular orbital (LUMO) orbital data provides an intuitive reflection of molecular reactivity. Reactivity and selectivity could be interpreted by comparing the HOMO and LUMO energy levels in the molecular docking results for the phenolic acid conformations, as well as the energy gap between them. The computational findings are presented in Fig. 11 and Table 6.

**Table 6:** Orbital energies of FA and CA.

Compound	*E*_HOMO_(a.u/mol)	*E*_LUMO_(a.u/mol)	Δ *E*_L-H_ (a.u/mol)
FA	−0.234	−0.177	0.057
CA	−0.295	−0.174	0.121

HOMO and LUMO are collectively referred to as the frontier orbitals. The difference in energy levels between the lowest unoccupied molecular orbital (*E*_LUMO_) and the highest occupied molecular orbital (*E*_HOMO_), denoted as Δ*E*_L-H_, represents the energy required for an electron to transition from the ground state to the excited state. This can be used to measure how readily a molecule can be excited [38].

The Δ*E*_L-H_ for FA was lower than that of CA, signifying that the electrons within the reactive functional groups of FA were more readily excitable (Table 6). The higher the HOMO energy level, the easier it is for electrons to be donated to an acceptor, and the lower the LUMO energy level, the more readily it could accept electrons. The HOMO and LUMO of FA were predominantly localized in the -C3-O1-CH_3_ region, indicating a substantial electron cloud density and a correspondingly strong reactive potential (Fig. 11). Conversely, the HOMO and LUMO of CA were distributed uniformly across the benzene ring's conjugated system, resulting in an even distribution of the electron cloud and consequently a lesser overall molecular reactivity compared with FA. Quantum mechanical computations provided a clear illustration of how the transformation of the hydroxyl group at the C3 position into a methoxy group in the structure of phenolic acids altered the distribution of the orbitals and affected overall molecular reactivity [39].

## 4 Discussion

The traditional Chinese medicine Sanguisorbae Radix boasts an abundance of wild resources, a venerable history of medicinal use, and a trove of folkloric records, classifying it as a medicinally edible substance. It is known for a variety of therapeutic effects, including hemostasis, anti-inflammatory, antibacterial, antitumor properties, and immune system enhancement. Scholars from around the world have conducted extensive research on Sanguisorbae Radix, yet systematic and in-depth studies on its chemical components and pharmacological properties are lacking. Most pharmacological activity experiments have been conducted on crude extract level, and studies exploring the correlation between chemical constituents and pharmacological activity are scarce and lack depth. Systematic molecular level research into its active substance basis and mechanisms of action are particularly lacking, which limits its clinical application [6,8].

SR exhibits commendable hemostatic properties, primarily attributable to its tannin and phenolic acid content [40]. Preliminary studies conducted by our research group indicated that the hemostatic efficacy of *Sanguisorba officinalis* is by enhanced post-processing, a phenomenon significantly associated with the increased concentration of phenolic acids following carbonization [41]. Previous studies suggests that the hemostatic agents within *Sanguisorba officinalis* can precipitate proteins into water-insoluble macromolecules that coagulate on the mucosal surface, thereby facilitating hemostasis. However, the precise mechanisms of the interaction between these compounds and proteins remain elusive [42].

Accordingly, this study selected the main hemostatic active components of SR (CA, FA, and their 1:1 mixture) as subjects for the investigation of the molecular mechanism underlying the hemostatic effect of SR.

Luo, Qi, Li and Akbari conducted experiments on the coagulative properties of CA and FA. Their research demonstrated that both substances were capable of reducing the time required for blood coagulation, prolonging the prothrombin time, augmenting the activity of thrombin, and facilitating platelet aggregation. The procoagulant effect was more pronounced with ferulic acid than with cinnamic acid. When the two were combined, the activity promoting coagulation was further enhanced [39,[43], [44], [45]].

This study, utilizing network pharmacological screening, identified the potential universal hemostatic target of SR phenolic acid as HSA. We investigated the interactions between SR phenolic acid and HSA by employing a combination of SDS-PAGE, multiple spectral methods, molecular docking, MD simulations, and quantum chemistry.

The SDS-PAGE method indicated that the binding of SR to HSA in plasma reduced its migration rate, with a retardation sequence of 1:1 > FA > CA. The UV and fluorescence spectroscopy experiments revealed that the phenolic acids bound to the Suldow site I on HSA, in close proximity to the tryptophan residues. The quenching constants for fluorescence followed the same order of 1:1 > FA > CA. CD spectroscopy showed that the interaction between phenolic acids and HSA induced a conformational change in HSA, characterized by a decrease in the α-helix content and an increase in β-turn and β-sheet structures. The extent of change followed the order: 1:1 > FA > CA. Molecular docking provided quantitative calculations of the binding of the phenolic acids to HSA, with the binding energies following the same order: 1:1 > FA > CA. Both the molecular docking and experimental results consistently demonstrated that the binding strength of the phenolic acids with HSA was in the order: 1:1 > FA > CA.

The order of the binding strength aligns with the reported sequence of their coagulation-promoting activities in the literature, suggesting that the stronger the binding of small molecules to HSA, the better the coagulation-promoting activity. This confirms that HSA is indeed the effective protein responsible for the hemostatic properties of SR phenolic acids.

MD were employed to visually analyze the molecular mechanisms of interaction. They demonstrated that the binding between the small molecules and HSA is stable, with no evidence of small molecule escape, and consistent with the docking results. This confirms the stability of the optimal binding model between HSA and small molecules, indicating that their interaction was not the result of transient molecular collisions but rather a sustained, stable state of interaction. The key amino acids involved in the binding of HSA with FA were Arg222 and Arg257, while for CA, it is Arg222. The stronger binding of FA compared to CA can be attributed to its methoxy group at position C3, which allows for greater torsional flexibility and molecular pliability. Additionally, the methoxy group's hydrophobic nature is more potent than the hydroxyl group at CA's position C3, thus facilitating easier insertion into the hydrophobic active pocket of HSA and enabling more hydrogen bond formation. Consequently, the active site of phenylpropanoid phenolic acids for hemostatic activity is likely at position C3, with the side chain attached at this position being the key functional group for hemostasis and directly correlating with its procoagulant activity. The stronger the hydrophilicity of the substituent group at this position, the less likely it is to bind with HSA, resulting in diminished hemostatic activity.

The binding affinity of the mixture with HSA surpassed that of the individual monomers, indicating a synergistic enhancing effect present in SR involving FA and CA. This synergism arises as the compounds, upon combine and, bind to different active regions within HSA's Suldow site I without competitive interference, thereby producing an additive effect. The increase in the number of hydrogen bonds and the expansion of the active binding pocket led to a stronger composite binding compared to the individual components. Arg222, Arg257 and His142 are the key amino acids instrumental in SR's hemostatic function. Specifically, His142 of HSA are critical residues for the synergistic enhancement of SR. The hydroxyl group at C3 of CA, along with the oxygen groups at C9 of FA, are the key sites contributing to the synergistic enhancement by the combination of FA and CA.

Quantum chemistry elucidates the mechanism of action between the two entities from an atomic perspective. The robust reactive activity of FA is attributed to the localization of its HOMO and LUMO primarily in the side chain region of C3-O1-CH_3_. Conversely, CA exhibited a higher degree of conjugation with an even distribution of HOMO and LUMO. This suggests that the extent of molecular conjugation was correlated with its binding activity. The higher the degree of conjugation, the weaker the reactive activity.

Literature has shown that the subdomain IIA of HSA contains two clusters of polaramino acids: an inner cluster with amino acid residues Tyr150, His242, and Arg257, and an external one, in the entrance of the pocket, with amino acids Lys195, Lys199, Arg218, and Arg222 [46]. The binding anchor sites of monounsaturated and polyunsaturated fatty acids to HSA were Tyr150, His242, Arg257 and Lys195, Lys199, Arg218, Arg222 [47,48], respectively, and the binding anchor sites of the synthetic NBD-C12 fatty acid synthesized NBD-C12 fatty acids were Ser202, Trp214, Lys199, His242 [49]. The CA and FA systems studied in this manuscript have binding residues ranging from Trp214 to Ala291. The overlapping anchor sites of CA and FA with unsaturated fatty acids are Lys199 and Arg222, while there is no overlapping anchor with NBD-C12. The reason may be that CA and FA are phenylpropanoid aromatic carboxylic acids with aromatic rings, with phenolic hydroxyl and carboxyl groups in their structures, while unsaturated fatty acids contain carboxyl groups, and the structure of NBD-C12 contains a polar heterocyclic group in addition to carboxyl groups [50], and these structural differences may lead to the differences in the binding anchor sites.In this study, we mainly discovered the effects of the phenolic hydroxyl groups of the side chains in the structure of FA and CA on the binding site of HSA. In the next phase, we plan to expand a series of studies on the interaction of aromatic ring structures and polar heterocyclic structures introduced by their modifications with HSA binding.

## 5 Conclusion

The study findings suggest that the binding of phenolic acids from SR to HSA constitutes one of the molecular mechanisms behind the hemostatic effects of SR. The capacity to bind was directly linked to the type and position of substituent groups, indicating that structural modifications could enhance its hemostatic activity. The synergistic effect of the SR phenolic acid mixture on HSA indicates that these results and research strategies could be applied to the screening for the optimal composition of hemostatic active ingredients in SR, thereby improving its clinical utility. Additionally, the study results provided insights into the molecular design and development of hemostatic traditional Chinese medicines and offer theoretical guidance for the binding patterns of phenylpropanoid phenolic drugs with the carrier protein HSA, as well as their transportation and pharmacological efficacy.

## Data availability

The data that support the findings of this study are available from the corresponding author, upon reasonable request.

## Ethics statements

This study was reviewed and approved by Nanjing University of Chinese Medicine under Chinese Guidelines of Laboratory Animal Care and Use in Research (Ministry of Health, Beijing, China) with the approval number:AEWC 20220105183, dated 20220105.

## CRediT authorship contribution statement

**Fei Xu:** Writing – original draft, Project administration, Data curation, Conceptualization. **YuQing Shen:** Supervision, Resources. **ZhiQiang Pan:** Software, Methodology, Investigation. **Xuan Zhou:** Validation, Software, Methodology. **Wei Gu:** Formal analysis. **Jie Dong:** Validation, Investigation. **Shaoping Yin:** Visualization, Resources. **ShengJin Liu:** Writing – review & editing, Supervision. **Ming Xu:** Resources, Investigation. **Baoduan Chen:** Investigation, Formal analysis.

## Declaration of competing interest

The authors declare that they have no financial interests or personal relationships that could have appeared to influence the work reported in the present study.
